# Placenta Prævia

**Published:** 1862-12

**Authors:** R. B. Treat

**Affiliations:** Janesville, Wis.


### ARTICLE XL.

### PLACENTA PRÆVIA.

By DR. R. B. TREAT, of Janesville, Wis.

It having been my ill-fortune to be called to attend two cases of Placenta Prævia, within the past two months; and, having pursued a different plan in the treatment of them than I have formerly done, I have thought proper to report them for publication.

Case 1.—Mrs. Craven, aged about 35 years, of a vigorous constitution, and the mother of several children. She was at full time, and labor commenced in the morning, having previously had several attacks of hemorrhage, as she informed me. She had been under the care of an Irish midwife, rough and ignorant; and the flooding gradually increased as the os uteri became dilated, until evening, when it became so excessive that syncope ensued, and I was sent for in haste. I lost no time in getting to the patient; found that she had rallied somewhat, but the pulse was still small, feeble, and rapid. There was but little prospect of saving the woman's life, let the case be what it might.

I immediately made an examination per vagina, and found the placenta presenting. The vagina was filled with coagula. The os was well dilated; and I restrained the hemorrhage by introducing the hand, until I could prepare some ergot. I also sent for Dr. E. F. Dodge to assist me. I then gave freely of the ergot, and carefully perforated the placenta in the centre, passed my hand to the fundus of the uterus, ruptured the membranes, secured the feet of the child, brought them down and delivered through the opening in it. Dr. Dodge made steady and firm pressure over the uterus, at the same time following it down as the child was expelled. The placenta came away immediately, and the flooding ceased. The uterus contracted well, from the pressure and ergot administered; and no further hemorrhage occurred during her recovery. In fact, very little blood was lost during the time intervening, from my arrival and the delivery. A firm bandage with compress was neatly applied, and a nourishing diet ordered; she was also directed to remain quietly in bed for a few days. Her recovery was rapid and perfect.

Case 2.—Mrs. B. a young married woman, aged 26, with feeble constitution. She was six months' advanced, and had been under the care of Dr. Dodge some four weeks. The hemorrhage was quite excessive at times, as she informed me; but as the os uteri was undilated, and her strength good, the usual remedies for uterine hemorrhage were resorted to, which controlled it for the time being. Finally, at the end of the sixth month, labor pains came on: the os yielded, and the flooding became so profuse, that it was necessary to interfere to save the life of the mother. An examination confirmed Dr. DODGE'S previous diagnosis. As in the other case, the placenta was located centrally over the os.

Chloroform and ether was administered, and when fully under its influence, I proceeded slowly and carefully to dilate the vagina; which after a while was accomplished. Then inserting my fingers in the usual way into the os, gradually dilated it, and introduced my hand, perforated the placenta as in the previous case, ruptured the membranes high up, secured the feet and delivered by turning as before through the placenta; which immediately followed the delivery of the child. No flooding took place after the introduction of the hand, and she soon rallied from the operation, with much less prostration than usual in such cases; attributable, I concluded, to the use of the anesthetic. In this case peritoneal inflammation followed which was successfully combated by Dr. DODGE; who treated the case afterwards. She made a good recovery. In both of these cases the children were lost; owing in the one case to the premature labor, and in the other from so large a portion of the placenta having been so long detached. It is perhaps necessary to add that my hand and arm were well oiled, previous to its introduction, or that the operation should not be undertaken without the use of chloroform.

In reporting these cases for publication, it is with a desire to call the attention of the profession to the ease and rapidity by which delivery may be accomplished by this mode of procedure; as well as to add my testimony to the revival of a method so pertinaceously urged and opposed by the earlier authors—on obstetric practice. It has been my fortune to have four cases of complete, and six partial placental presentations. Of the four complete, delivery was accomplished in two of them by detaching the placenta at the side, a method by far more tedious and dangerous in my estimation. One of the cases died a few minutes after delivery, from the fact that the attending physician used the tampoon without the knowledge of the presentation; and leaving it until a fatal hemorrhage had taken place, before I was called. In the other case, I saved both mother and child, but the recovery was long delayed. Of the other class of cases, the labor was completed in three by rupturing the membranes, and bringing the head down by the placental mass; and the other three by turning,—all successful. When the practitioner is so unfortunate as to meet a case of this kind, he is in duty bound to watch it with the most untiring solicitude, and never resort to the tampoon, unless to give time to send for counsel, (which should always be had, if possible,) and not then, if he possesses the requisite knowledge, and courage to proceed with an operation that requires so much skill and perseverance, to save the life of a patient placed in so much jeopardy by one of the worst complications to be met with in obstetric practice.

